# Segmental Pigmentation Disorder: A Case Report of Hypopigmented Patch

**DOI:** 10.7759/cureus.33692

**Published:** 2023-01-12

**Authors:** Ammar Bakhsh, Khalid Al Hawsawi, Ola Y Bahadur, Ruba Z Alotaibi, Mohammed F Bondagji, Sarah M Fageeh

**Affiliations:** 1 Medicine and Surgery, Umm Al-Qura University, Makkah, SAU; 2 Dermatology, King Abdulaziz Hospital, Makkah, SAU; 3 Medicine and Surgery, King Saud bin Abdulaziz University for Health Sciences, Jeddah, SAU

**Keywords:** mosaicism, segmental vitiligo, segmental pigmentation disorder, vitiligo, early childhood

## Abstract

A segmental pigmentation disorder (SPD) is a form of pigmentary mosaicism. SPD is a hypo- or hyperpigmented patch that has a segmental pattern. A 16-year-old male with an insignificant past medical history presented with symptomless, slowly progressive skin lesions since early childhood.

Skin examination revealed well-demarcated, non-scaling, hypopigmented patches on the right upper extremity. A similar spot was located on his right shoulder. Wood’s lamp examination showed no enhancement. Differential diagnoses included segmental pigmentation disorder and segmental vitiligo (SV). A skin biopsy was obtained, which revealed normal findings. Based on the above clinicopathological findings, a diagnosis of segmental pigmentation disorder was made. The patient did not receive any treatment but was reassured that he did not have vitiligo.

## Introduction

Segmental pigmentation disorder (SPD) is a form of pigmentary mosaicism. It is characterized by hyperpigmented or, less commonly, hypopigmented patches that occur in a segmental pattern with a midline demarcation and less distinct serrated lateral margins. SPD has a block-like, flag-like, or checkerboard-like shape.

SPD was first described by Metzker and colleagues in 1983 and has several names, including pigmentary mosaicism, segmental pigmentation abnormality, segmental nevus depigmentosus, giant café-au-lait macule, and patterned dyspigmentation [1,2]. Although the pathogenesis of SPD is multifaceted, genetic analysis has detected postzygotic gene alterations or chromosomal anomalies [3]. This condition begins to manifest during childhood. Moreover, any site can be affected, with the trunk being the most common site and more often ventral than dorsal [3]. SPD is usually not associated with extracutaneous features [2]. Histopathologically, the hypopigmented lesions of SPD show reduced or normal numbers of melanocytes [2]. Here, we report a 16-year-old male who had a segmental hypopigmented patch since early childhood, necessitating the differentiation between segmental vitiligo (SV) and SPD.

## Case presentation

A 16-year-old male with an insignificant past medical history presented with asymptomatic and slowly progressive skin lesions since early childhood. Past medical history, medication history, and review of systems were all unremarkable. Similar cases were not detected in the family, and the parents were not consanguine.

**Figure 1:**
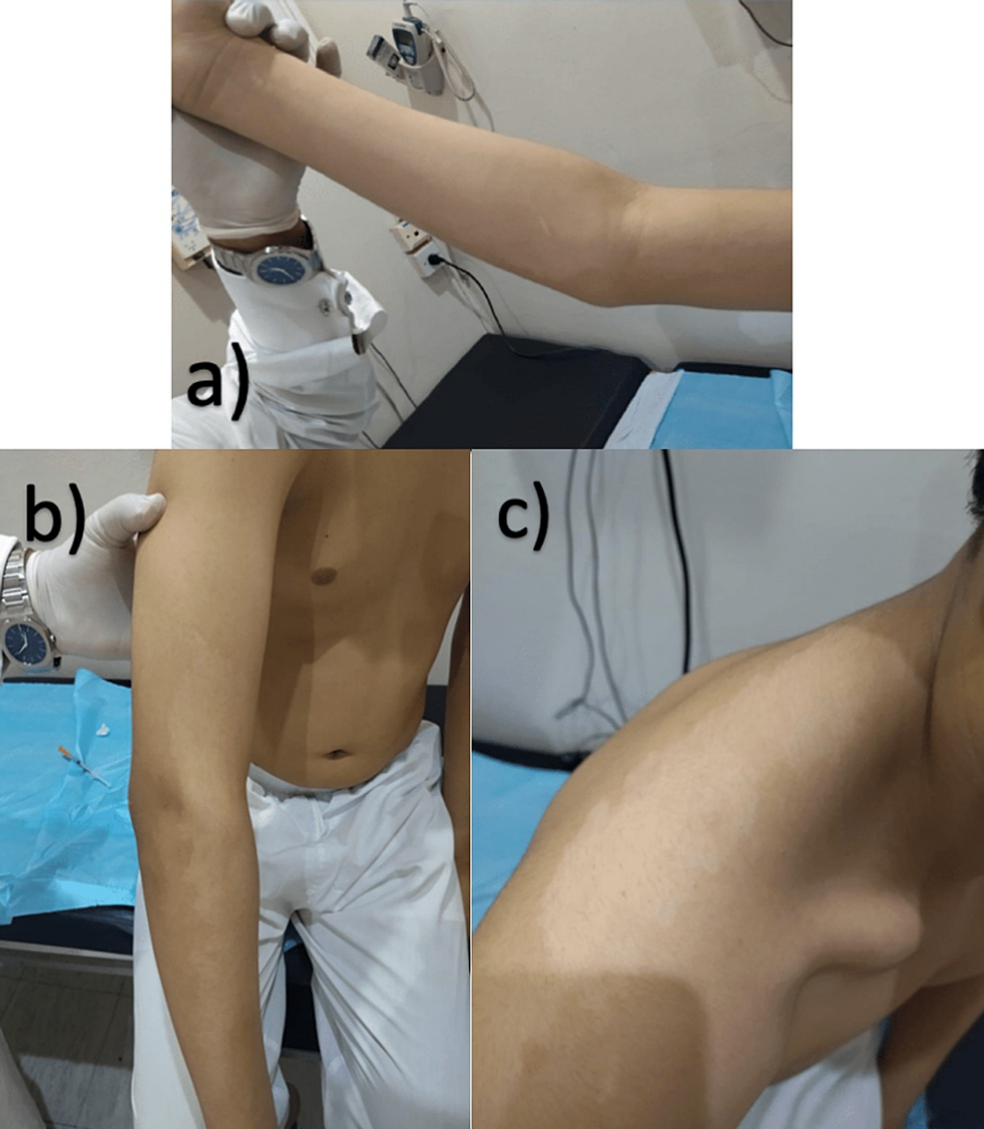
One large, well-demarcated with irregular borders, non-scaly, hypopigmented patches in the right forearm (a and b) and right shoulder (c).

**Figure 2:**
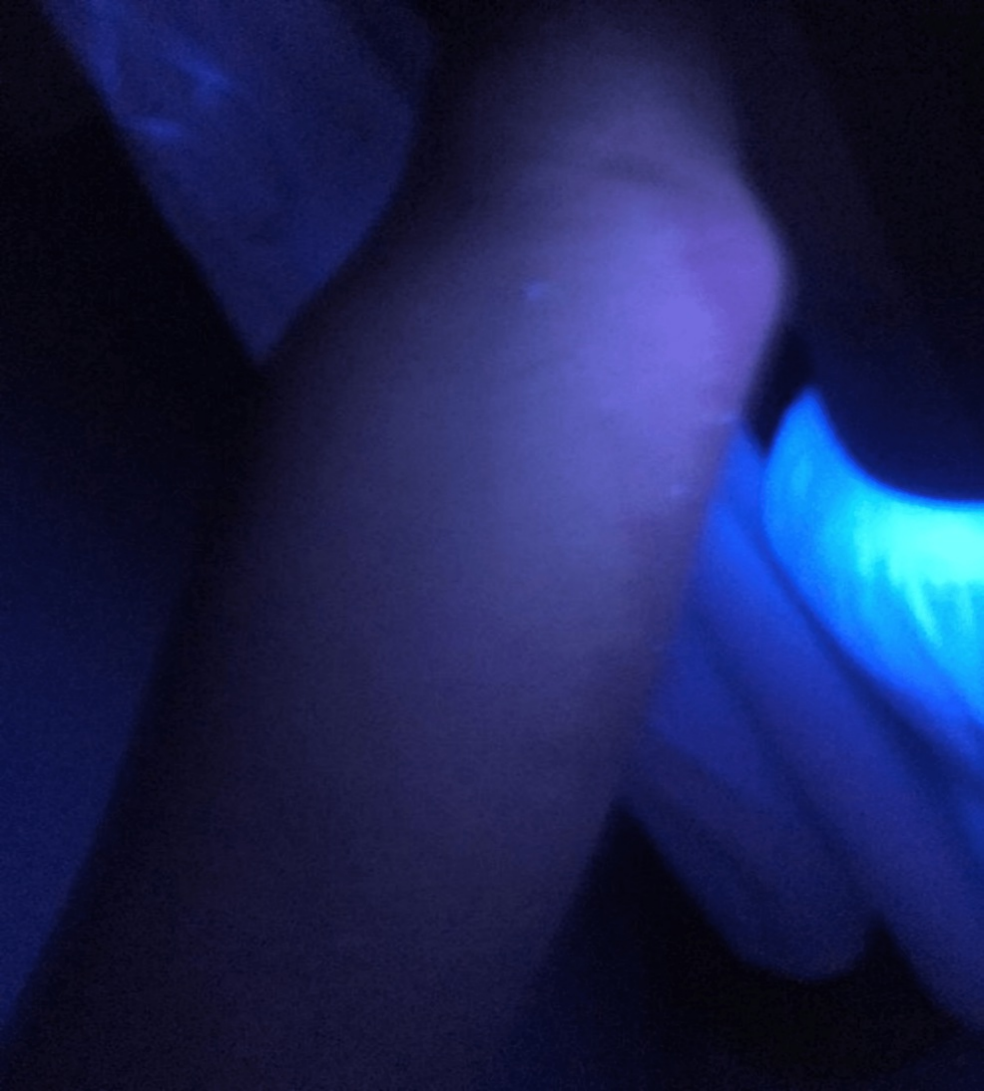
No enhancement of hypopigmentation on Wood’s lamp examination.

Skin examination revealed a wide 24 × 14 cm, well-demarcated with irregular borders, non-scaling, hypopigmented patch covering most of his right upper extremity. A similar spot was located on his right shoulder (Figure 1). Differential diagnoses included SPD, SV, and hypopigmented mycosis fungoides. On examination with Wood’s lamp, no enhancement of hypopigmentation was noted (Figure 2). A skin specimen was obtained through a biopsy, which showed normal skin. No other pathological findings were noted. The dermoscopic examination was not done based on the above clinicopathological findings and Wood’s lamp examination; the diagnosis of SPD was made. The patient did not receive any treatment but was reassured that he did not have vitiligo.

## Discussion

The diagnosis of SPD was made clinically. The main equivalent diagnoses included SV, pigmented demarcation lines type A, and hypopigmented mycosis fungoides. SV is characterized by depigmented skin patches rather than hypopigmented patches, which are usually unilateral and often affect the face [4]. While most people have only one portion, a small minority of patients have two or more segments in SV [5]. The absence of enhancement of hypopigmentation under Wood’s lamp in our patient suggested the diagnosis of SPD. Although early vitiligo may not show enhancement under Wood’s lamp, our patient had the lesions for more than 10 years. In pigmentary demarcation lines type A, there is a characteristic sharp vertical line on the anterolateral surface of the upper arm. However, our patient had a well-demarcated patch with irregular borders on his right upper extremity.

SPD frequently occurs on the trunk and is limited to one segment. Our patient had two segments, on the shoulder and upper extremity, which are rare locations for SPD [6]. Another differential diagnosis that was essential to exclude in our patient was hypopigmented mycosis fungoides, a disease that persists for decades. The absence of scales or xerosis, segmental distribution, and histopathological findings ruled out hypopigmented mycosis fungoides in our patient [7]. In addition, histopathologically, both SPD and SV show normal skin. Ultrastructurally, vitiligo shows the absence of melanocytes, although some may be present, while SPD shows melanocytes [8].

The identification of the underlying genetic defect does not usually affect treatment. However, genetic analysis should be considered in any patient with extracutaneous manifestations as it provides a more accurate diagnosis [9].

Treatment of SPD includes the management of the extracutaneous manifestations; however, no extracutaneous manifestations were present in our patient. Hypopigmentation is usually persistent and does not respond to laser treatment. Although spontaneous fading of the hyperpigmented type of SPD has been shown, it has not been reported for the hypopigmented type [10].

## Conclusions

Although the hypopigmented type of SPD is rare, it should be considered in the differential diagnoses of SV. We should increase the awareness of this entity. The absence of the enhancement of hypopigmentation under Wood’s lamp in a segmental hypopigmented patch in a child who had been present for several years supported the diagnosis of SPD. SPD on the extremities, as in our patient, is rare.
